# Authors’ Response to Peer Reviews of “Determinants of Periodic Health Examination Uptake: Insights From a Jordanian Cross-Sectional Study”

**DOI:** 10.2196/71528

**Published:** 2025-02-05

**Authors:** Abdul Aziz Tayoun

**Affiliations:** 1 School of Medicine, Department of Family and Community Medicine, Jordan University, Qween Rania Street, Amman, 11942, Jordan, 962 778435800

**Keywords:** periodic health examination, PHE, preventive health services, routine health checkups, Jordan, cross-sectional study

*This is the author’s response to peer-review reports for “Determinants of Periodic Health Examination Uptake: Insights From a Jordanian Cross-Sectional Study.”*

## Round 1 Review

### Anonymous [1]

*The following items were noted in this paper [2].*

- *Periodic health examination (PHE) uptake: Only 27.1% of participants underwent a PHE in the last 2 years.*
- *Predictors: Significant predictors include recent visits to a primary health care facility, monthly income, and knowledge about PHEs and preventive health measures.*
- *Nonsignificant factors: Gender, marital status, smoking status, and BMI did not show a significant association with PHE uptake.*

#### Strengths

- *Comprehensive analysis: The study employs a robust methodology, combining descriptive, inferential, and multivariate statistical techniques to provide a thorough understanding of PHE uptake.*
- *Significant predictors identified: Key factors influencing PHE uptake were identified, offering valuable insights for health care providers and policy makers.*
- *First of its kind in Jordan: This study fills a gap in existing knowledge by being the first to investigate PHE uptake in Jordan.*

#### Negative Points and Areas for Improvement

##### Cross-Sectional Design

- *Limitation: The study’s design limits the ability to establish causality.*
- *Improvement: Future research could benefit from a longitudinal approach to better establish causal relationships between the identified predictors and PHE uptake.*

**Response:** We acknowledge the limitation of the cross-sectional design in establishing causality and have highlighted this in the Discussion section, suggesting future longitudinal studies.

##### Convenience Sampling

- *Limitation: This method may introduce selection bias, and the online survey format may lead to measurement bias.*
- *Improvement: Employing a more randomized and stratified sampling method could enhance the representativeness and validity of the findings.*

**Response:** We have clarified the rationale for using convenience sampling due to resource constraints and have suggested more randomized methods for future studies.

##### Limited Generalizability

- *Limitation: Results may not be generalizable to populations outside of Jordan or those not included in the sample.*
- *Improvement: Expanding the study to include diverse populations and different geographic regions would provide a more comprehensive understanding of PHE uptake.*

**Response:** We understand the concern regarding generalizability. However, as the study aimed to estimate PHE uptake and its determinants specifically in Jordan, the focus on this population was intentional. For future research, we recommend conducting multinational studies, particularly in Arab countries, or performing systematic reviews or meta-analyses to obtain results that can be generalized beyond Jordan.

##### Survey Instrument

- *Limitation: The questionnaire’s comprehensiveness and relevance to the Jordanian context might not have been fully ensured.*
- *Improvement: Pretesting the survey with a larger and more varied group, followed by adjustments based on feedback, could improve its applicability and accuracy.*

**Response:** We have taken steps to improve the relevance and comprehensiveness of the questionnaire by pretesting it and incorporating feedback.

##### Behavioral Factors

- *Limitation: The study did not find a relationship between behavioral factors and PHE uptake, which contradicts findings in other contexts.*
- *Improvement: A more detailed investigation into cultural and societal influences on health behaviors in Jordan is needed to clarify these results.*

**Response:** We agree that further investigation into cultural and societal influences on health behaviors in Jordan is needed and have discussed this in the manuscript.

##### English Language and Clarity

- *Limitation: The manuscript contains some grammatical errors and awkward phrasings, which can detract from its readability.*
- *Improvement: A thorough review and editing for language and clarity by a native English speaker or professional editor would enhance the manuscript’s quality.*

**Response:** The manuscript has undergone a thorough review and editing process to enhance its readability and clarity.

Thank you for these excellent comments. We have thoroughly reviewed and integrated your suggestions into the main manuscript.

### Reviewer AV [3]

#### Specific Comments

##### Major Comments

*1. In this manuscript, write in detail about the data collection procedure.*

**Response:** The data collection process was reviewed in detail. Please refer to the Methodology section and note that the questionnaire has been added as an appendix (see Multimedia Appendix 1).

*2. Why was a convenience sampling technique employed?*

**Response:** A convenience sampling technique was employed due to resource constraints, as the study was not funded and was conducted by a single author. This has been mentioned in the Methodology section.

*3. “All collected data are treated with strict confidentiality.” Some language corrections are required.*

**Response:** We have rephrased the Ethical Consideration section to improve clarity and accuracy.

##### Minor Comments

*There are a lot of formatting issues; many things seem copied and pasted.*

**Response:** We have addressed the formatting issues to ensure consistency and clarity throughout the document.

## Abbreviations

PHE: periodic health examination
